# Impact of pH, Temperature and Exogenous Proteins on Aspartic Peptidase Secretion in *Candida auris* and the *Candida haemulonii* Species Complex

**DOI:** 10.3390/pathogens14090873

**Published:** 2025-09-02

**Authors:** Gabriel C. Silva, Pedro F. Barbosa, Lívia S. Ramos, Marta H. Branquinha, André L. S. Santos

**Affiliations:** 1 Laboratório de Estudos Avançados de Microrganismos Emergentes e Resistentes (LEAMER), Departamento de Microbiologia Geral, Instituto de Microbiologia Paulo de Góes (IMPG), Universidade Federal do Rio de Janeiro (UFRJ), Rio de Janeiro 21941-901, RJ, Brazil; campsgabriel04@gmail.com (G.C.S.); pedrofdbarbosa@gmail.com (P.F.B.); liviaramos2@yahoo.com.br (L.S.R.); 2 Universidade Estadual do Rio de Janeiro (UERJ), Rio de Janeiro 20559-900, RJ, Brazil; 3 Programa de Pós-Graduação em Ciências (Microbiologia), Instituto de Microbiologia Paulo de Góes (IMPG), Universidade Federal do Rio de Janeiro (UFRJ), Rio de Janeiro 21941-901, RJ, Brazil; 4 Programa de Pós-Graduação em Bioquímica, Instituto de Química, Universidade Federal do Rio de Janeiro (UFRJ), Rio de Janeiro 21941-909, RJ, Brazil; 5 Rede Micologia RJ—FAPERJ, Rio de Janeiro 21941-901, RJ, Brazil

**Keywords:** *Candida auris*, *Candida haemulonii* complex, aspartic peptidases, virulence, Saps, growth conditions

## Abstract

*Candida* species commonly secrete aspartic peptidases (Saps), which are virulence factors involved in nutrient acquisition, colonization, tissue invasion, immune evasion and host adaptation. However, the regulation of Sap production remains poorly characterized in emerging, widespread and multidrug-resistant members of the *Candida haemulonii* clade (*C. auris*, *C. haemulonii*, *C. haemulonii* var. *vulnera* and *C. duobushaemulonii*). This study investigated the influence of temperature, pH and protein substrate on Sap production using bloodstream isolates of the *C. haemulonii* clade. Sap activity was initially assessed using the enzyme coefficient (Pz) in fungal cells grown on yeast carbon base (YCB) agar supplemented with bovine serum albumin (BSA) to determine optimal conditions for enzymatic production. *C. auris* and *C. duobushaemulonii* exhibited the highest Sap activity at 96 h, pH 4.0–5.0, and 37 °C, whereas *C. haemulonii* and *C. haemulonii* var. *vulnera* displayed more variable and isolate-dependent profiles. Sap production was markedly suppressed at pH 6.0. The addition of pepstatin A, an inhibitor of aspartic peptidases, abolished Sap activity and impaired fungal growth in a dose-dependent manner, confirming both the enzymatic identity and its critical role in nitrogen acquisition. Conversely, YCB supplemented with an inorganic nitrogen source (ammonium sulfate) supported fungal growth but did not induce Sap production. To explore substrate specificity, YCB was supplemented with a panel of proteins. Serum albumins (bovine and human) induced the highest Sap production, followed by globulin, gelatin, hemoglobin, collagen and immunoglobulin G, while elastin and mucin elicited the lowest Sap production. Isolate-specific preferences for protein substrates were observed. Finally, fluorometric assays using a Sap-specific fluorogenic peptide substrate confirmed the presence of Sap activity in cell-free supernatants, which was consistently and entirely blocked by pepstatin A. These findings highlight inter- and intraspecies variability in Sap regulation among *C. haemulonii* clade, stressing the critical roles of substrate availability, pH and temperature in shaping fungal adaptation to host environments.

## 1. Introduction

In recent years, research on fungal infections has expanded substantially, highlighting their growing recognition as a global threat to human health [1]. This heightened interest stems from several factors, including the presence of different types of fungi within the human microbiota, where they typically exist as commensal organisms. However, under certain conditions, such as microbiota dysbiosis or immune system impairment, these fungi can transition to pathogenic behavior, triggering infectious processes in the human host [2]. Consequently, certain populations are at higher risk of opportunistic fungal infections, which exploit weakened immune defenses [3]. High-risk groups include individuals undergoing chemotherapy, those receiving broad-spectrum antimicrobials, organ transplant recipients, patients with autoimmune diseases and individuals at the extremes of age [1,2,3].

*Candida* species have attracted growing attention due to the increasing incidence of invasive infections and the alarmingly high associated mortality rates, which range from 46% to 75%, depending on the species, host conditions and clinical context [4]. Among them, *C. albicans*, *C. glabrata (Nakaseomyces glabratus*), *C. tropicalis*, *C. parapsilosis* and *C. krusei (Pichia kudriavzevii*) are the most frequently associated with infections worldwide. These *Candida* species contribute to a wide range of clinical manifestations, from superficial mucosal infections to life-threatening systemic candidiasis [1,4]. Notably, species within the *C. haemulonii* complex (*C. haemulonii sensu stricto*, *C. duobushaemulonii* and *C. haemulonii* var. *vulnera*; current known as *Candidozyma haemuli*, *Candidozyma duobushaemuli* and *Candidozyma haemuli* var. *vulneris*, respectively), along with *C. auris (Candidozyma auris)*, a closely related phylogenetic species, collectively form the *C. haemulonii* clade. Species within the *C. haemulonii* clade have been increasingly reported across all continents, with varying incidence rates. Although they currently represent a smaller proportion of fungal infections compared to other *Candida* species, their emergence as multidrug-resistant pathogens poses a growing clinical concern. Notably, their intrinsic resistance to commonly used antifungal agents, particularly polyenes (e.g., amphotericin B) and azoles (e.g., fluconazole, itraconazole and voriconazole), significantly limits treatment options for deep-seated infections. This resistance is frequently linked to increased mortality rates, particularly in healthcare settings, where these yeasts can persist, spread and cause outbreaks [5,6].

The ability of *Candida* species to establish infections across diverse anatomical sites is largely attributed to their remarkable adaptability and array of virulence attributes. These factors include biofilm formation, adhesins and the secretion of a broad spectrum of hydrolytic enzymes, all extensively studied for their pivotal roles in pathogenicity [7]. Among these, hydrolytic enzymes are particularly crucial, as they degrade key host molecules—such as lipids and proteins—facilitating essential stages of infection, including adhesion, nutrient acquisition, tissue invasion, colonization and evasion [8]. This multifaceted virulence strategy enables *Candida* species to thrive in different host environments and evade immune responses. In this context, secreted aspartic peptidases (Saps) play a pivotal role in *Candida* pathogenicity by degrading host proteins within the mucosa and extracellular matrix, which are critical barriers against infection. By breaking down these structures, Saps facilitate pathogen nutrition, adhesion, invasion and dissemination into the bloodstream, potentially leading to systemic infections [9]. Beyond their proteolytic function, Saps contribute to immune evasion and infection persistence. Their enzymatic activity modulates host immune responses by impairing immune cell functions, thereby enabling the fungus to evade detection and clearance. Moreover, Saps promote biofilm formation by modifying fungal cell surfaces, exposing adhesion-promoting proteins that strengthen biofilm architecture and enhance resistance to host defenses and antifungal treatments [9].

Given the multifaceted roles of Saps in *Candida* pathogenicity, understanding how environmental factors regulate their production remains a critical focus of current research. Like other virulence factors, Sap activity is finely regulated by physicochemical environmental conditions, which can vary significantly across different host niches. Despite their clinical relevance, Sap production remains poorly characterized in emerging, multidrug-resistant species of the *C. haemulonii* clade. To address this gap in the literature, this study investigated the effects of temperature, pH and protein substrate availability on Sap production in representative species of the *C. haemulonii* clade (*C. auris*, *C. haemulonii sensu stricto*, *C. duobushaemulonii* and *C. haemulonii* var. *vulnera*). The analyzed isolates, recovered from bloodstream infections, displayed resistance to conventional antifungals, particularly fluconazole and/or amphotericin B. By elucidating how these environmental parameters influence Sap activity, the study aims to deepen our understanding of virulence regulation in these emerging multidrug-resistant yeasts.

## 2. Material and Methods

### 2.1. Microorganisms

This study analyzed eight clinical isolates recovered from blood samples (Table 1), including four *C. auris* isolates (Ca885, Ca446, Ca383 and Ca384) and four isolates from the *C. haemulonii* complex (LIPCh11, LIPCh12, LIPCh13 and LIPCh23). The *C. auris* isolates Ca885 and Ca446 were obtained from the Centro de Investigaciones Microbiológicas del Cesar (CIMCE) and the Proteomic and Human Mycosis Investigation Unit at Universidad Javeriana, Colombia, respectively [10]. *C. auris* isolates Ca383 and Ca384 were sourced from the Centers for Disease Control and Prevention (CDC), USA [11]. Regarding the *C. haemulonii* complex, *C. haemulonii* var. *vulnera* LIPCh11 and *C. haemulonii* LIPCh12 were acquired from the Instituto Nacional do Câncer (INCA, Rio de Janeiro), Brazil, while *C. duobushaemulonii* isolates LIPCh13 and LIPCh23 were obtained from the Diagnósticos da América S.A. (DASA) microbial collection, Brazil [12].

### 2.2. Growth Conditions

The fungal isolates were stored in Sabouraud culture medium supplemented with 20% glycerol and preserved at −80 °C. Additionally, fungi were maintained on Sabouraud agar plates for preservation and handling before the experiments. For analysis, fungal cells were cultured in yeast carbon base (YCB; HiMedia, Mumbai, India) supplemented with 0.1% bovine serum albumin (BSA) (Sigma-Aldrich, St. Louis, MO, USA) under constant shaking (200 rpm) at 37 °C for 48 h, following the protocol described by Ramos et al. [8]. After cultivation, yeast cell counts were determined using a Neubauer chamber [13].

### 2.3. Plate Testing Assay

Aspartic peptidase activity was assessed using a plate assay prepared with 2.35% YCB agar supplemented with 0.1% BSA, which is a well-established condition known to induce Sap production in various *Candida* species [14], including those from the *C. haemulonii* species complex [5,12] and *C. auris* [5,11]. Before inoculation, yeast cell suspensions were prepared at a concentration of 10^6^ cells per mL in PBS. A 10 μL aliquot of each suspension was inoculated onto agar plates. The plates were incubated under different conditions, including pHs (4.0, 5.0 and 6.0) and temperatures (37 °C to simulate human body temperature and 28 °C to represent environmental conditions). Colony growth and the formation of BSA degradation halos around the fungal colonies were monitored and measured daily over a 96 h period. Aspartic peptidase activity was semi-quantified using the enzyme coefficient (Pz), calculated as the ratio between the colony diameter and the total diameter of the colony plus its corresponding degradation halo [15]. Lower Pz values corresponded to higher enzymatic activity, while higher Pz values indicated reduced activity. Based on Pz values, enzymatic activity was classified into four categories: a Pz equal to 1 indicated no enzymatic activity (negative); values between 0.999 and 0.700 were classified as weak activity; values between 0.699 and 0.400 represented moderate activity; and values between 0.399 and 0.100 were indicative of excellent enzymatic activity [15,16]. To confirm that BSA degradation was mediated by an aspartic-type peptidase, fungal cells were inoculated onto YCB-BSA agar plates supplemented with increasing concentrations of pepstatin A (0.1, 1 and 10 μM), a classical and specific inhibitor of aspartic peptidases. Fungal growth and Sap production were then compared to those observed on YCB-BSA plates without the peptidase inhibitor. In parallel, YCB medium was supplemented with either an organic (0.1% BSA) or an inorganic (0.1% ammonium sulfate) nitrogen source to evaluate the influence of nitrogen availability on fungal growth and Sap production. As expected, the marked reduction in both fungal growth and Sap activity in the presence of pepstatin A confirmed the involvement of aspartic peptidases in BSA hydrolysis.

### 2.4. Modulation of Sap Production in Response to Different Protein Substrates

After determining the optimal temperature, incubation time and pH conditions for Sap production in clinical isolates of the *C. haemulonii* clade, the influence of different protein substrates on enzyme activity was evaluated. For this purpose, YCB agar was supplemented with 0.1% of various proteins, including BSA, human serum albumin (HSA), mucin from porcine stomach, elastin from human lung, gelatin from porcine skin, hemoglobin from bovine blood, immunoglobulin G (IgG) from human serum, collagen from bovine Achilles tendon and gamma globulin from bovine blood (all obtained from Sigma-Aldrich, St. Louis, MO, USA). The experiment was conducted as described in Section 2.3, with colony growth and degradation halos measured to calculate Pz values after 96 h. However, for some protein substrates, the hydrolytic halos were not clearly visible. To enhance visualization of proteolytic activity, agar plates were stained with Amido Black (0.1% *w*/*v* in 45% methanol and 10% acetic acid) after 96 h of incubation. This method, using amido black in plates containing YCB medium supplemented with BSA, exhibited regions where aspartic peptidase activity occurred with a light blue coloration, contrasting with the dark blue background, indicating protein substrate degradation [17]. This approach improved the precision of the analysis, enabling a more detailed characterization of the enzymatic activity of the tested isolates. A comparison was also made between Pz activity on unstained plates and those stained with amido black. To further characterize enzymatic activity, a scoring system was developed, assigning each isolate a total score based on its performance across all substrates. Based on the Pz score, excellent activity received 3 points, moderate activity 2 points and weak activity 1 point. This approach provided a more detailed analysis of Sap production and its potential role in the infectious process.

### 2.5. Quantification of Sap Activity Using Fluorogenic Peptide Substrate

To confirm Sap production by clinical isolates of the *C. haemulonii* clade, fungal cultures (10^6^ cells) were incubated for 96 h in YCB broth supplemented with 0.1% of each previously described protein substrate. After incubation, cultures were centrifuged (4000× *g*, 5 min, 4 °C), and the supernatants were filtered through 0.22 μm membranes (Millipore, São Paulo, SP, Brazil). Protein concentrations were determined using the method described by Lowry et al. [18], using BSA as the standard. Enzymatic activity was measured using a cathepsin D-specific fluorogenic peptide substrate [7-methoxycoumarin-4-acetyl-Gly-Lys-Pro-Ile-Leu-Phe-Phe-Arg-Leu-Lys(DNP)-D-Arg-amide] (Sigma-Aldrich, St. Louis, MO, USA) [13,19,20]. Reactions were initiated by adding 2 μM of substrate to 50 μg of total protein from each culture supernatant, diluted in 50 mM sodium acetate buffer (pH 4.0), at a final volume of 100 μL [13]. Substrate cleavage was monitored continuously for 1 h at 37 °C using a spectrofluorometer (SpectraMax Gemini XPS, Molecular Devices, San Jose, CA, USA), with excitation at 328 nm and emission at 393 nm. To assess specificity, reactions were carried out both in the absence and presence of 10 μM pepstatin A. Supernatants without the peptidase inhibitor served as positive controls. Enzymatic activity was expressed in arbitrary fluorescence units (AFUs) [16].

### 2.6. Statistical Analyzes

All experiments were performed in triplicate and independently repeated at least three times to ensure the reliability of the results and minimize variability. Statistical analyses were carried out using Student’s *t*-test for comparisons between two groups and one-way analysis of variance (ANOVA) for comparisons between three or more groups. Differences were considered statistically significant when *p* ≤ 0.05. All statistical analyses were conducted using GraphPad Prism 8 software.

## 3. Results

### 3.1. Modulation of Sap Production Across Different pHs and Temperatures over Time

**Figure 1:**
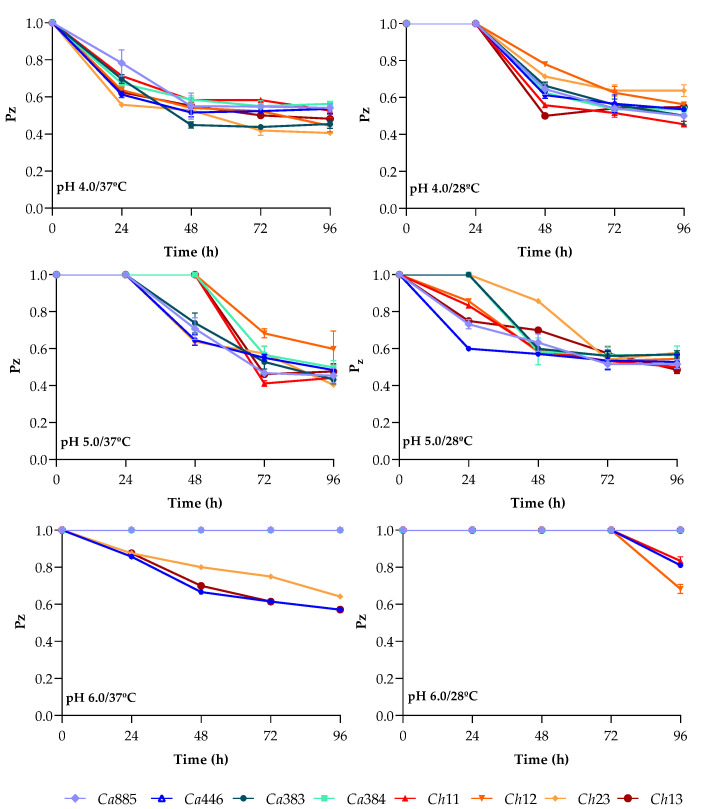
Aspartic peptidase production by clinical isolates of the *C. haemulonii* clade under varying pH, temperature and incubation time conditions. Fungi were inoculated onto the surface of YCB-BSA medium adjusted to pH 4.0, 5.0 or 6.0, and incubated at either 37 °C or 28 °C for up to 96 h. At 24, 48, 72 and 96 h, Sap activity was expressed by Pz value, defined as the ratio of the colony diameter to the total diameter of the colony plus its surrounding degradation halo. The fungal isolates tested included *C. auris* 885 (Ca885), *C. auris* 446 (Ca446), *C. auris* 383 (Ca383), *C. auris* 384 (Ca384), *C. haemulonii* var. *vulnera* 11 (Ch11), *C. haemulonii* 12 (Ch12), *C. duobushaemulonii* 13 (Ch13) and *C. duobushaemulonii* 23 (Ch23). Data are expressed as the mean ± standard deviation from three independent experiments, each conducted in triplicate.

The experimental conditions for aspartic peptidase detection resulted in varying levels of enzymatic activity among clinical isolates of the *C. haemulonii* clade, as illustrated in Figure 1. Notably, pH 4.0 at 37 °C was the only condition that supported detectable enzymatic activity in all isolates after 24 h of incubation, demonstrating greater consistency compared to other conditions. Among the tested parameters, pH 5.0 at 37 °C yielded the lowest Pz values for most isolates—indicative of the highest Sap activity—particularly between 72 and 96 h of incubation. The following Pz values were recorded at this condition: *C. auris* 383 (0.433 ± 0.020), *C. auris* 885 (0.454 ± 0.032), *C. auris* 446 (0.484 ± 0.011), *C. auris* 384 (0.498 ± 0.029), *C. duobushaemulonii* Ch23 (0.404 ± 0.005), *C. duobushaemulonii* Ch13 (0.461 ± 0.033) and *C. haemulonii* var. *vulnera* Ch11 (0.411 ± 0.008). An exception was observed for *C. haemulonii* Ch12, which exhibited its highest activity (Pz = 0.444 ± 0.022) at pH 4.0 and 37 °C after 96 h.

When comparing Sap activity at pH 4.0 and pH 5.0 (both at 37 °C) over 96 h, both conditions supported moderate activity across nearly all isolates, with no statistically significant differences in Pz values (Figure 1). In contrast, at pH 6.0, Sap production displayed distinct kinetics, failing to support enzymatic activity in most fungal isolates (Figure 1). This inhibitory effect was more pronounced at 28 °C than at 37 °C. Likewise, at 28 °C, both pH 4.0 and 5.0 resulted in significantly lower Sap production across all time points compared to the corresponding conditions at 37 °C (Figure 1). These observations reveal clear differences in the kinetic behavior of aspartic peptidase activity, suggesting isolate-specific variations in Sap regulation under different physicochemical conditions.

The aforementioned findings suggest that an incubation temperature of 37 °C, especially at pH 5.0 and with a 96 h incubation period, optimized Sap production in the tested *C. haemulonii* clade species. Therefore, these parameters were chosen for the subsequent experiments.

### 3.2. Inhibition of Sap Production by Aspartic Peptidase Inhibitor

**Figure 2:**
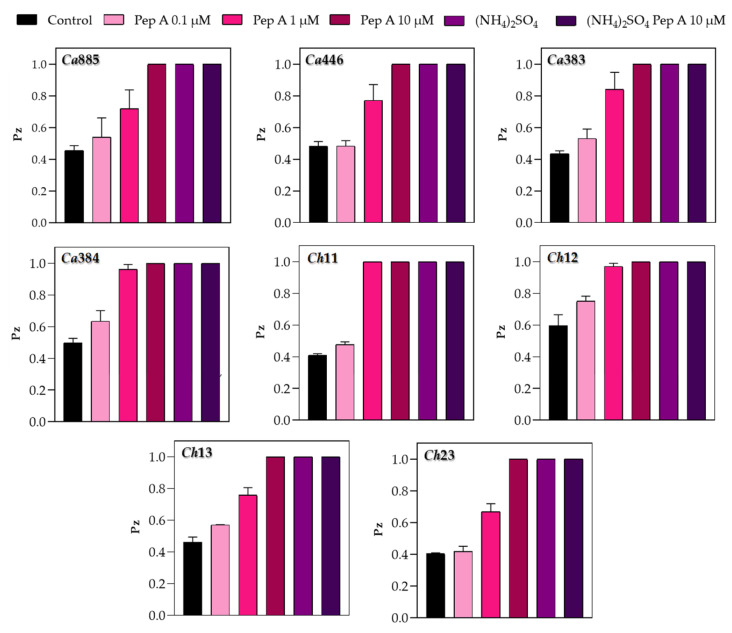
Impact of the prototypical aspartic peptidase inhibitor pepstatin A on Sap production by clinical isolates of the *C. haemulonii* clade cultivated with organic (BSA) and inorganic ((NH_4_)_2_SO_4_) nitrogen sources. A standardized inoculum (10^6^ cells) was spotted onto YCB-BSA agar plates (pH 5.0) containing different concentrations of pepstatin A (PepA at 0.1, 1 and 10 μM) or no inhibitor. Plates were incubated at 37 °C for 96 h. Sap activity was assessed by the presence or absence of proteolytic halos surrounding the colonies, indicating inhibition of aspartic peptidase activity in the presence of the inhibitor. In parallel, isolates were cultivated on YCB medium supplemented with ammonium sulfate ((NH_4_)_2_SO_4_) as the sole nitrogen source, with or without pepstatin A, to evaluate the effect of the inhibitor under inorganic nitrogen conditions. The isolates tested included *C. auris* 885 (Ca885), *C. auris* 446 (Ca446), *C. auris* 383 (Ca383), *C. auris* 384 (Ca384), *C. haemulonii* var. *vulnera* 11 (Ch11), *C. haemulonii* 12 (Ch12), *C. duobushaemulonii* 13 (Ch13) and *C. duobushaemulonii* 23 (Ch23).

**Figure 3:**
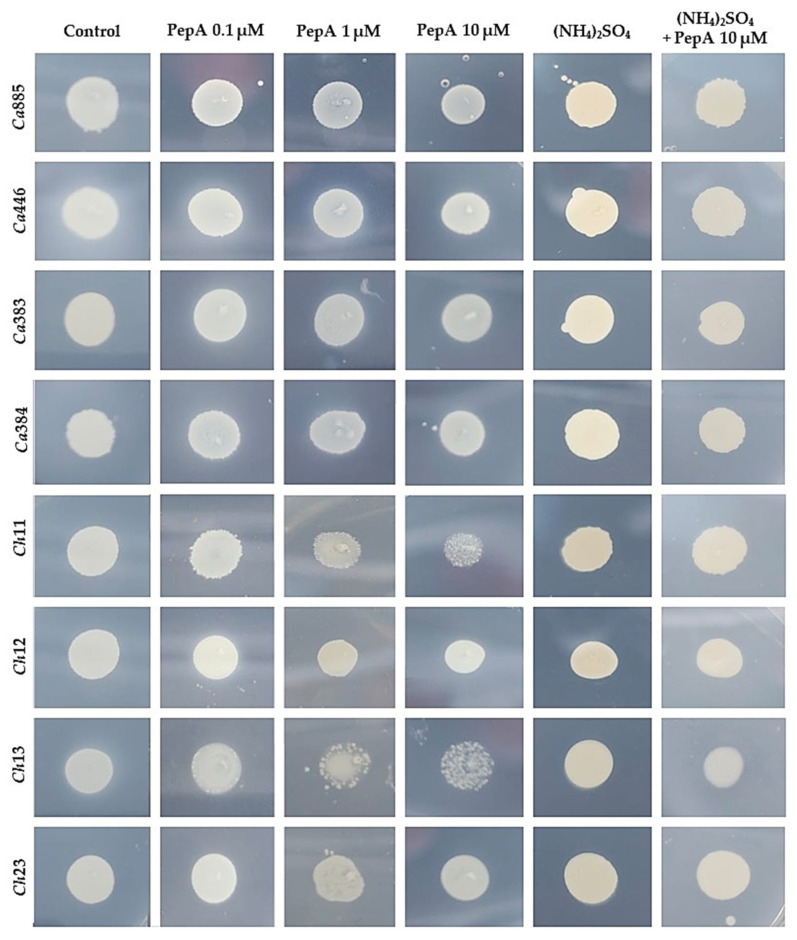
Impact of the prototypical aspartic peptidase inhibitor pepstatin A on the growth of clinical isolates of the *C. haemulonii* clade cultivated with organic (BSA) and inorganic ((NH_4_)_2_SO_4_) nitrogen sources. A standardized inoculum (10^6^ cells) was spotted onto YCB-BSA agar plates (pH 5.0) containing different concentrations of pepstatin A (PepA at 0.1, 1 and 10 μM) or no inhibitor. Plates were incubated at 37 °C for 96 h and fungal growth was assessed based on colony formation. In parallel, isolates were also grown on YCB medium supplemented with ammonium sulfate ((NH_4_)_2_SO_4_) as the sole nitrogen source, with or without pepstatin A, to evaluate the effect of the inhibitor under inorganic nitrogen conditions. The isolates tested included *C. auris* 885 (Ca885), *C. auris* 446 (Ca446), *C. auris* 383 (Ca383), *C. auris* 384 (Ca384), *C. haemulonii* var. *vulnera* 11 (Ch11), *C. haemulonii* 12 (Ch12), *C. duobushaemulonii* 13 (Ch13) and *C. duobushaemulonii* 23 (Ch23).

To confirm that the proteolytic activity observed in plate assays was specifically attributable to the secretion of aspartic peptidases, the typical inhibitor pepstatin A was incorporated into the YCB-BSA medium. Fungal cells were then inoculated onto the surface of the medium, and both growth and Sap production were monitored. The reduction in proteolytic halos observed in the presence of pepstatin A occurred in a dose-dependent manner, strongly supporting the conclusion that the enzymatic activity was mediated by aspartic-type peptidases (Figure 2). In addition, a reduction in fungal colony size, varying in extent depending on the isolate, was also observed under these conditions (Figure 3). This variation suggests isolate-specific differences in the reliance on Sap-mediated protein degradation for optimal growth, further highlighting the functional diversity of Sap activity within the *C. haemulonii* clade. Moreover, the fungal colony reduction likely reflects impaired fungal growth due to the inability to efficiently acquire nitrogen, as the inhibition of Sap activity prevents the proteolytic cleavage of albumin, the unique nitrogen source in this medium. Corroborating these findings, supplementation of YCB medium with an inorganic nitrogen source (ammonium sulfate) supported full fungal growth but did not induce Sap production (Figure 2 and Figure 3).

### 3.3. Modulation of Sap Production by Different Protein Substrates

**Figure 4:**
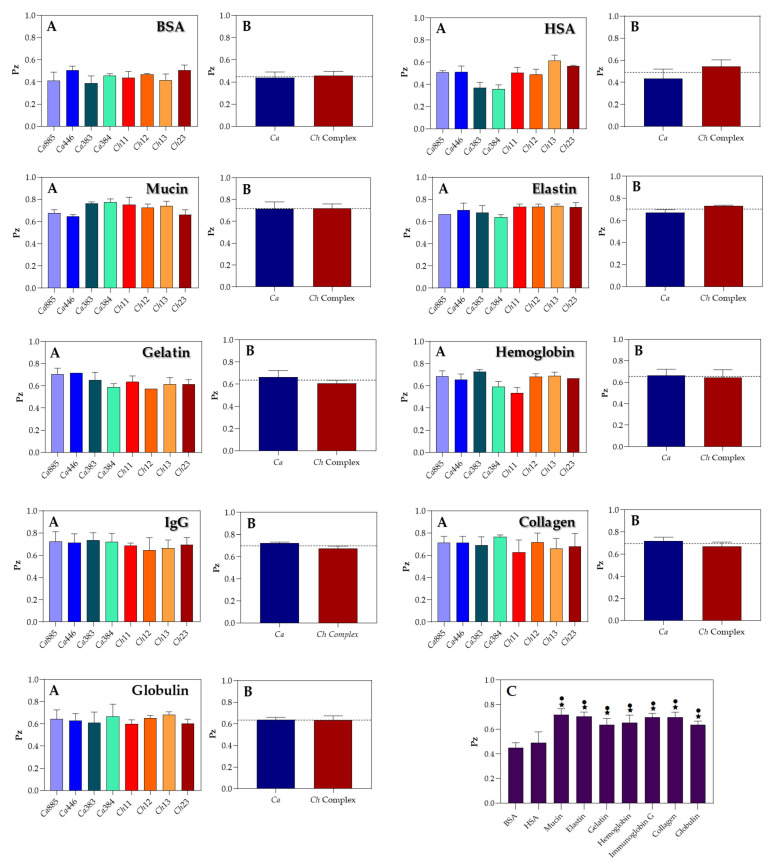
Sap detection in clinical isolates of the *C. haemulonii* clade cultured in aspartic peptidase-inducing medium supplemented with various protein substrates. A standardized inoculum (10^6^ cells) was applied to YCB agar plates supplemented with 0.1% of individual proteins (bovine serum albumin—BSA, human serum albumin—HSA, mucin, elastin, gelatin, hemoglobin, immunoglobulin G, collagen and globulin) adjusted to pH 5.0. Plates were incubated at 37 °C for 96 h and subsequently stained with Amido Black to enhance visualization of proteolytic halos. Sap activity was quantified by calculating the Pz value for each isolate (**A**). Mean Pz values were also calculated for the two taxonomic groups: *C. auris* and the *C. haemulonii* species complex (**B**). The overall mean Pz value for all fungal isolates is indicated by a dotted line in panels (**B**,**C**) to facilitate comparison across different protein substrates. Symbols indicate statistically significant differences (*p* < 0.001) according to Tukey’s multiple comparisons test, as follows: (★) denotes significant differences between BSA and all other proteins except HSA; (•) indicates significant differences between HSA and all other proteins, except BSA. The isolates tested included *C. auris* 885 (Ca885), *C. auris* 446 (Ca446), *C. auris* 383 (Ca383), *C. auris* 384 (Ca384), *C. haemulonii* var. *vulnera* 11 (Ch11), *C. haemulonii* 12 (Ch12), *C. duobushaemulonii* 13 (Ch13) and *C. duobushaemulonii* 23 (Ch23). Data are expressed as the mean ± standard deviation from three independent experiments, each conducted in triplicate.

In this set of experiments, Sap production by *C. haemulonii* clade species was evaluated by incubating fungal isolates in YCB medium supplemented with various proteinaceous sources. Overall, Sap activity ranged from weak to moderate, depending on the protein used. Notably, albumins (BSA and HSA) were the only substrates that consistently induced moderate to strong activity across all isolates, as reflected by their lowest mean Pz values (Figure 4, panels A). This response was particularly pronounced in *C. auris* isolates 383 and 384, both obtained from the CDC-USA, which exhibited the highest Sap activity under the tested conditions (Figure 4, panels A). In contrast, for the other tested proteins, no significant differences in Sap production levels were observed among the isolates, suggesting a broadly uniform enzymatic response regardless of the protein source (Figure 4, panels A).

To identify the most effective protein inducers of Sap production in the *C. haemulonii* clade, we calculated the mean Pz values for each test protein across all fungal isolates. The overall mean Sap activity is represented by a dotted line in Figure 4 (panels B), with the following average Pz values observed: BSA (0.448 ± 0.041), HSA (0.490 ± 0.083), globulin (0.636 ± 0.029), gelatin (0.637 ± 0.048), hemoglobin (0.654 ± 0.058), collagen (0.697 ± 0.040), IgG (0.699 ± 0.028), elastin (0.703 ± 0.035) and mucin (0.717 ± 0.046). These results indicate that most proteins supported moderate Sap activity among the isolates. The only exceptions were mucin and elastin, which exhibited mean Pz values above the threshold for moderate activity, thus being classified as weak inducers of Sap production. Furthermore, when the analysis was stratified by taxonomic group—comparing *C. auris* isolates with those from the *C. haemulonii* species complex—the mean enzymatic activity for each group was assessed based on Pz values across the different protein supplements (Figure 4, panels B). This comparative analysis revealed no statistically significant differences in Sap activity between the two groups for any of the tested proteins.

Notably, comparative analysis of mean Pz values across all tested proteins revealed a highly significant difference (*p* < 0.001) in Sap production when fungal cells were cultured in YCB medium supplemented with either BSA or HSA, compared to all other protein substrates (Figure 4, panel C). This finding underscores the potent Sap-inducing capacity of serum albumins. However, no statistically significant difference was observed between BSA and HSA, indicating that both proteins are equally effective in stimulating aspartic peptidase production in isolates from the *C. haemulonii* clade.

**Figure 5:**
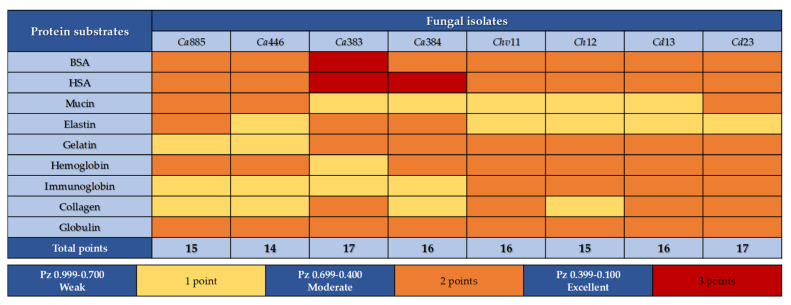
Heatmap illustrating protein degradation preferences among clinical isolates of the *C. haemulonii* clade. Based on the Pz values obtained for each isolate in response to different protein substrates (as shown in Figure 4), a semi-quantitative scoring system was developed to highlight the relative Sap production levels. For each protein tested, enzymatic activity was classified as strong (3 points), moderate (2 points) or weak (1 point), according to the corresponding Pz value. This scoring approach provides a comparative visualization of substrate-specific Sap induction patterns across isolates, revealing potential isolate-specific preferences and metabolic flexibility. The isolates tested included *C. auris* 885 (Ca885), *C. auris* 446 (Ca446), *C. auris* 383 (Ca383), *C. auris* 384 (Ca384), *C. haemulonii* var. *vulnera* 11 (Ch11), *C. haemulonii* 12 (Ch12), *C. duobushaemulonii* 13 (Ch13) and *C. duobushaemulonii* 23 (Ch23).

A scoring system was established to quantify Sap production by each fungal isolate belonging to the *C. haemulonii* clade based on the protein supplement used. Enzymatic activity was classified according to Pz values: weak (1 point), moderate (2 points) and strong (3 points). A total Sap activity score was calculated for each isolate by summing the individual scores across all nine tested proteins, with a maximum possible score of 27 points (i.e., strong activity for all substrates). The analysis showed that final Sap activity scores among the isolates varied between 14 and 17 points (Figure 5), indicating a relatively narrow range of variability among isolates. These results suggest a conserved proteolytic capacity within the *C. haemulonii* clade and highlight their shared ability to utilize a broad spectrum of proteinaceous substrates, which may contribute to their adaptability and persistence in diverse host environments [21,22,23].

### 3.4. Measurement of Sap Activity Via Fluorogenic Peptide-Based Assay

**Figure 6:**
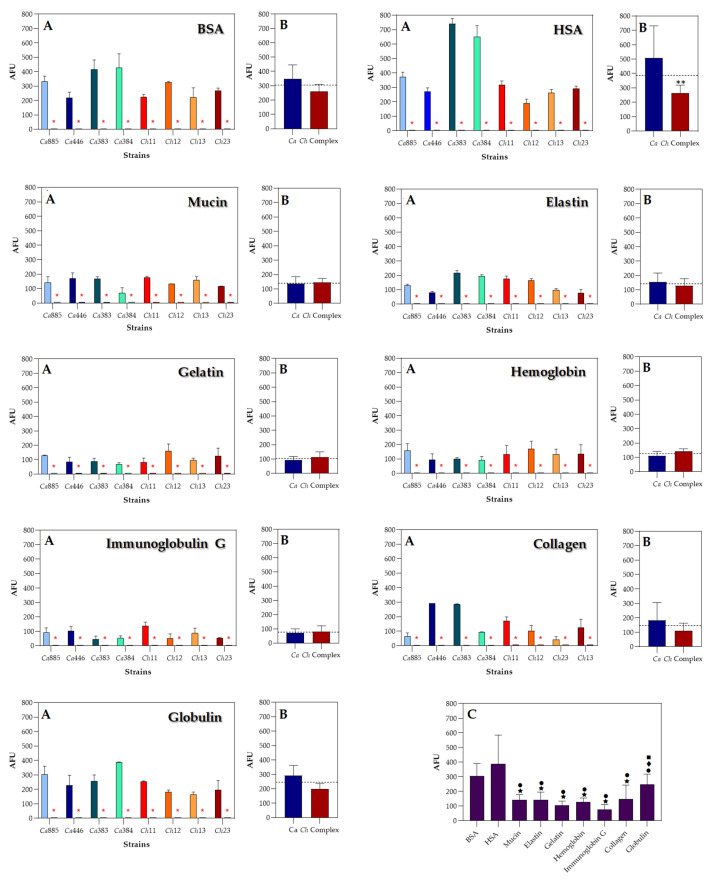
Quantification of Sap activity in clinical isolates of the *C. haemulonii* clade cultured in aspartic peptidase-inducing medium supplemented with various protein substrates. Fungal isolates were grown in YCB broth containing 0.1% of different protein supplements. After 96 h, culture supernatants were collected, filtered and used to measure Sap activity using a cathepsin D-specific fluorogenic peptide substrate selective for aspartic-type peptidases. Enzymatic reactions were carried out by incubating 2 μM of substrate with 50 μg of fungal supernatant in 50 mM sodium acetate buffer (pH 5.0) at 37 °C for 1 h. Fluorescence was continuously monitored (excitation: 328 nm; emission: 393 nm), and the 24 min time point was selected for analysis as it corresponded to the peak of substrate hydrolysis. Pepstatin A (10 μM), a classical aspartic peptidase inhibitor, was used to confirm the specificity of the enzymatic activity. Results are expressed in arbitrary fluorescence units (AFUs) for each isolate (**A**). A statistically significant difference was observed between non-treated and pepstatin A-treated systems for all isolates (*, *p* < 0.05). The mean AFU values were also calculated for the two taxonomic groups: *C. auris* and the *C. haemulonii* species complex (**B**). A statistically significant difference was observed between the two phylogenetic groups for HSA-induced Sap activity (**, *p* < 0.05). The overall mean AFU value for all fungal isolates is indicated by a dotted line in panels (**B**,**C**) to facilitate comparison across different protein substrates. Symbols indicate statistically significant differences (*p* < 0.05) according to Tukey’s multiple comparisons test, as follows: (★) denotes significant differences between BSA and all other proteins except HSA and globulin; (•) indicates significant differences between HSA and all other proteins, except BSA; (♦) indicates significant difference between gelatin and globulin; and (■) indicates significant difference between immunoglobulin G and globulin. The isolates tested included *C. auris* 885 (Ca885), *C. auris* 446 (Ca446), *C. auris* 383 (Ca383), *C. auris* 384 (Ca384), *C. haemulonii* var. *vulnera* 11 (Ch11), *C. haemulonii* 12 (Ch12), *C. duobushaemulonii* 13 (Ch13) and *C. duobushaemulonii* 23 (Ch23). Data are expressed as the mean ± standard deviation from three independent experiments, each conducted in triplicate.

Sap activity was quantified in the cell-free supernatants of *C. haemulonii* clade isolates using a cathepsin D-specific fluorogenic peptide substrate. The results revealed notable variations in enzymatic activity depending on both the supplemented protein source and the fungal isolate (Figure 6, panels A). For instance, among the tested proteins, albumins consistently induced the highest levels of Sap secretion across all fungal isolates, with a markedly pronounced effect in two *C. auris* isolates (Ca383 and Ca384). These findings closely mirrored those from the agar plate assays, with both *C. auris* isolates exhibiting the highest levels of substrate hydrolysis, further reinforcing the potent Sap-inducing capacity of albumin-rich environments. Overall, the average Sap activity across all fungal isolates for each protein substrate was as follows: HSA (386.5 ± 186.1 AFU), BSA (304.5 ± 79.8 AFU), globulin (245.8 ± 64.6 AFU), collagen (146.9 ± 89.9 AFU), mucin (142.0 ± 34.0 AFU), hemoglobin (126.1 ± 27.5 AFU), gelatin (103.4 ± 29.0 AFU) and IgG (76.9 ± 30.4 AFU) (Figure 6, panels B). Furthermore, when grouped by taxonomic classification, *C. auris* isolates exhibited significantly higher Sap activity (*p* < 0.05) compared to isolates from the *C. haemulonii* species complex in response to HSA. Although *C. auris* isolates also showed consistently higher mean Sap activity for BSA, elastin, collagen and globulin, as well as the *C. haemulonii* species complex demonstrated higher mean activity for mucin, gelatin, hemoglobin and IgG, none of these differences reached statistical significance (Figure 6, panels B).

Remarkably, comparative analysis of mean AFU values across all tested proteins revealed a statistically significant increase in Sap production (*p* < 0.05) when fungal cells were cultured in YCB medium supplemented with either BSA or HSA, compared to nearly all other protein substrates (Figure 6, panel C). This reinforces the strong Sap-inducing potential of serum albumins. Furthermore, additional statistically significant differences were detected between globulin-supplemented cultures and those containing either gelatin or IgG, indicating that globulin more effectively stimulates Sap activity than these both protein sources (Figure 6, panel C).

## 4. Discussion

Several *Candida* species commonly exist as commensals on human mucosal surfaces like the skin, gastrointestinal and genitourinary tracts. However, changes in the host environment or host–pathogen interactions can shift them from harmless colonizers to opportunistic pathogens capable of invading tissues and causing infections in various sites [2,4]. This transition relies largely on virulence factors that promote adaptation, tissue invasion, immune evasion and persistence. Among these, secreted hydrolytic enzymes—especially aspartic peptidases (Saps)—are key in breaking down host barriers and facilitating infection [9]. Because Sap expression is highly influenced by external factors such as pH, temperature and nutrient availability, this study investigated how these conditions affect Sap production in clinical isolates of *C. auris* and species from the *C. haemulonii* complex (*C. haemulonii*, *C. haemulonii* var. *vulnera* and *C. duobushaemulonii*). Known collectively as the *C. haemulonii* clade, these emerging, multidrug-resistant fungi have become clinically important due to their persistence in healthcare settings and resistance to standard antifungals. This study provides a comprehensive assessment of how physicochemical cues regulate Sap production in these medically relevant and widespread non-*albicans Candida* species.

In the initial assays, Sap activity was assessed under different temperatures (37 °C and 28 °C) and pH levels (4.0, 5.0 and 6.0). The results consistently showed higher enzymatic activity at acidic pH, especially at 4.0 and 5.0, evidenced by lower Pz values. The marked reduction or absence of activity at pH 6.0 reinforces the pH-dependent nature of aspartic peptidase production and catalytic efficiency under more acidic conditions [24,25]. Although pH 5.0 yielded the highest activity for most isolates of C. *haemulonii* clade, the difference from pH 4.0 was not statistically significant, suggesting inter-isolate variability in pH responsiveness. This variability in pH responsiveness is consistent with previous observations across multiple *Candida* species. For instance, optimal Sap production in *C. albicans* typically occurs within the pH range of 3.5 to 5.5, whereas *C. parapsilosis* exhibits peak proteolytic activity at even more acidic conditions, between pH 2.0 and 4.0 [24,26,27,28]. In a classic study, Homma et al. [27] investigated the effect of medium pH on Sap synthesis in *C. albicans* and reported a complete lack of induction at neutral pH, regardless of BSA supplementation. These findings underscore the strong pH-dependency of Sap expression. Interestingly, in that work, the optimal pH for Sap induction varied according to the presence of BSA: an initial pH of 4.0 favored maximal enzyme production in BSA-supplemented media, whereas pH 5.0–5.5 was optimal in its absence. This shift suggests a dual modulatory role of pH, influencing both enzymatic activity and nutrient acquisition. In media containing BSA, Sap-mediated proteolysis is enhanced under acidic conditions (with catalytic optima between pH 3.5 and 4.0), which facilitates the liberation of peptides and amino acids, thereby enriching the nitrogen pool [29]. This acid-enhanced proteolytic response likely supports both fungal growth and efficient Sap expression, reflecting a finely tuned adaptation to environmental cues. The findings highlight the remarkable pH-dependent plasticity of *Candida* species, particularly their capacity to thrive in acidic niches. Notably, *C. haemulonii* clade isolates exhibit a pronounced adaptation to low-pH conditions, optimizing Sap production in environments that resemble host-associated sites, such as mucosal surfaces or inflamed tissues, where the local pH is often reduced [24,25,26,27,28]. This acidophilic profile likely contributes to their persistence and pathogenicity, especially in anatomical sites characterized by acidic microenvironments.

Temperature also significantly influenced Sap activity in *C. haemulonii* clade, with higher enzyme production consistently observed at 37 °C compared to 28 °C across all isolates. Although a slight delay in activity onset was noted, peak production generally occurred after prolonged incubation. These findings align with previous studies on *C. auris*, which also showed enhanced Sap production at 37 °C versus lower temperatures such as 25 °C [30,31]. Similar patterns have been reported for *C. parapsilosis*, *C. albicans* and *C. haemulonii*, where Sap activity increases near human body temperature [32,33]. This thermoregulated response likely represents an evolutionary adaptation that promotes fungal survival and virulence within the host. The correlation between peak enzymatic activity and physiological temperature suggests that thermal cues act as environmental signals for triggering virulence factor expression during infection. Although the underlying mechanisms remain incompletely understood, studies indicate a complex interplay of transcriptional and post-transcriptional regulation in response to temperature shifts [34,35]. Elucidating how thermal cues regulate Sap expression may offer critical insights into the molecular mechanisms underlying fungal pathogenicity and could inform the development of targeted antifungal strategies. Temperature is a key environmental signal that not only influences gene expression but also modulates fungal physiology and virulence. The ability of *Candida* species to grow and express virulence-associated factors, such as Saps, at human body temperature is essential for successful colonization, persistence and progression to infection. This thermotolerance, coupled with the induction of pathogenic traits in response to host-like conditions, underscores the importance of temperature as both a permissive and regulatory factor in the establishment of *Candida*-related diseases.

It is well known that the synthesis and secretion of Sap2 by *C. albicans* can be tightly regulated by the presence of exogenous proteins, such as albumin and hemoglobin, when these are provided as the sole nitrogen source in a defined medium [28,32]. This regulation appears to operate via a positive feedback mechanism, wherein the proteolytic degradation of these high-molecular-weight proteins generates peptide fragments that, in turn, serve as signals to upregulate Sap2 expression. These peptides likely act either directly through nutrient-sensing pathways or indirectly by modulating transcriptional regulators of *SAP* genes, thereby amplifying peptidase production. Such a mechanism ensures efficient nitrogen acquisition while reinforcing the fungus’s capacity to adapt to protein-rich host niches, particularly in bloodstream and tissue environments [28,32]. Supporting this mechanism, our findings show that the addition of pepstatin A to YCB-BSA medium inhibited Sap activity and significantly reduced fungal growth. This effect was likely due to impaired nitrogen acquisition, as blocking albumin degradation limits the release of peptides and amino acids essential for fungal metabolism. Collectively, these findings highlight the critical role of Saps not only in virulence, but also in supporting basic physiological processes such as nutrient assimilation and proliferation when preferred nitrogen sources are scarce.

To better replicate host-like conditions, in this study, we assessed Sap activity in response to various protein substrates commonly present at different anatomical sites. Among them, BSA and HSA consistently triggered moderate to strong enzymatic responses across most isolates of *C. haemulonii* clade, demonstrating their high Sap-inducing potential. Scoring analysis showed that the majority of isolates exhibited moderate activity for at least five out of nine tested proteins, indicating a broad but selective proteolytic profile. Notably, blood-derived proteins—especially albumins and globulin—elicited the highest Sap activity in both plate-based Pz assays and spectrofluorometric measurements, with *C. auris* isolates displaying the strongest responses. This preference aligns with their bloodstream origin and suggests an adaptation to plasma-rich environments, where these proteins serve as abundant nitrogen sources. These findings highlight how Sap production is not only influenced by pH and temperature, but also finely regulated by substrate availability, reflecting the fungi’s metabolic flexibility and environmental sensing during systemic infections. Except for IgG, which induced the lowest Sap production, other proteins like gelatin, hemoglobin, collagen, elastin and mucin yielded intermediate AFU values (approximately 105–150), indicating detectable yet lower enzymatic responses. Plate assays confirmed weak activity when mucin, elastin or collagen were the sole nitrogen sources, supporting the fluorometric data. Given that these substrates are key components of mucosal and connective tissues, their limited degradation suggests that bloodstream isolates may be less adapted to colonize or invade mucocutaneous surfaces. Instead, these fungi appear specialized for systemic environments, possibly reflecting a trade-off that prioritizes persistence and nutrient acquisition in the circulatory system over initial host barrier penetration.

The protein degradation profile exhibited by *C. haemulonii* clade isolates suggests a form of ecological specialization, likely shaped by selective pressures within the bloodstream. The observed preference for serum substrates, coupled with limited activity against mucosal or structural proteins, may reflect an evolutionary adaptation that prioritizes Sap functionality in plasma-like environments. In this context, a functional trade-off may emerge: enhanced proteolytic efficiency in the bloodstream could come at the expense of enzymatic versatility required for colonization of mucosal surfaces [36]. Such niche-specific enzymatic tuning is well documented in other fungal pathogens. For example, in glucose-rich environments like the bloodstream, *C. albicans* downregulates the glyoxylate cycle. However, under nutrient-limited conditions, such as those found inside macrophages, it reactivates this pathway, a key adaptation that supports both survival and virulence [36]. Similarly, *Cryptococcus neoformans* and *Aspergillus fumigatus* exhibit nitrogen and carbon catabolite repression, allowing them to selectively exploit available nutrients based on environmental cues, thereby optimizing metabolism in response to local conditions [37]. While these examples involve broader metabolic pathways, they illustrate the overarching principle of functional fine-tuning to specific host niches. Although comparable regulatory mechanisms remain largely uncharacterized in the *C. haemulonii* complex, the specialized Sap profile observed here may stem from similar processes. It is plausible that bloodstream-adapted strains express a narrower, serum-focused Sap repertoire, optimized for the metabolic constraints and substrate composition of this niche. In contrast, species such as *C. albicans* exhibit broader enzymatic plasticity, facilitated by the differential expression of multiple SAP isoforms that enable adaptation across a wide range of host environments. For example, *C. albicans* Sap6 is strongly upregulated in mucin-rich environments, contributing to epithelial adhesion and mucosal colonization [38], a mechanism not yet described in *C. haemulonii* species complex or *C. auris*. This disparity may reflect a downregulation or absence of mucin-targeting Saps in bloodstream-adapted species. Comparative proteolytic profiling of isolates derived from mucosal or cutaneous sites versus those from bloodstream infections may help clarify whether these patterns represent transient niche-specific expression or fixed, clade-level evolutionary adaptations. Such analyses could offer valuable insights into the ecological strategies that drive peptidase specialization and host tropism within the *C. haemulonii* clade.

The ability of *Candida* species to degrade a wide range of protein substrates enhances their pathogenic potential by promoting tissue invasion and colonization of various host sites. This enzymatic flexibility supports key virulence traits such as adhesion, nutrient acquisition, biofilm formation, immune evasion and host barrier penetration [39,40,41,42]. Barbosa et al. [3], for instance, demonstrated that *C. albicans* cultured in YCB medium supplemented with BSA or HSA showed increased expression of surface Sap1–3 antigens compared to cultures exposed to gelatin or IgG. Interestingly, those authors also reported strong antigen expression in the presence of mucin and hemoglobin—unlike the current study, where these proteins induced only weak enzymatic responses. Such differences may result from species-specific regulation or the anatomical origin of the isolates. Moreover, substrate-specific modulation of individual Sap isoenzymes may further explain the divergent findings [7,21,34]. These results illustrate the multifactorial control of Sap production, shaped by environmental inputs—such as nutrient type and pH—as well as intrinsic microbial factors, including genetic background, isoform expression and ecological adaptation. A deeper understanding of these regulatory networks is essential for unraveling the role of Saps in the virulence of emerging multidrug-resistant species forming the *C. haemulonii* clade.

Variations in Sap production and substrate-specific cleavage patterns likely reflect not only species-level regulatory differences but also isoenzyme-level specificity. The Sap family comprises multiple isoforms, each potentially specialized to degrade particular substrates or respond to distinct environmental signals. In *C. albicans*, for example, ten *SAP* genes (*SAP1*–*SAP10*) have been identified, encoding peptidases with diverse expression profiles and substrate affinities that contribute to the species’ broad ecological adaptability. In contrast, other *Candida* species possess fewer *SAP* paralogs, *C. tropicalis* encodes four, *C. dubliniensis* eight and *C. parapsilosis* only three [43], suggesting that gene family composition may significantly influence proteolytic potential and niche specialization. In *C. auris*, seven *SAP* homologs (*SAPA1–SAPA7*) have been identified, with *SAPA3* emerging as the most strongly expressed isoform under infection-relevant conditions [31]. It is plausible that only a subset of these isoforms is inducible in the presence of host substrates such as mucin or hemoglobin, potentially accounting for the limited or selective enzymatic activity observed in this study. Such specificity may indicate that bloodstream-adapted species have evolved a more restricted, yet functionally optimized Sap repertoire, tailored to the nutrient composition and immune pressures of that particular niche. These findings support the broader hypothesis that differences in *SAP* gene content and isoform regulation among *Candida* species are key determinants of their proteolytic capacity and host adaptation strategies. However, further molecular and functional characterization, including isoform-specific expression analysis and substrate profiling, is needed to elucidate the extent and implications of this specialization within the *C. haemulonii* clade.

Overall, our findings highlight the adaptive flexibility of Sap production in *C. auris* and members of the *C. haemulonii* complex in response to environmental cues resembling host conditions. This adaptability supports their persistence, tissue invasion and virulence in bloodstream infections. The modulation of enzymatic activity by factors such as substrate type, pH and temperature reflects a finely tuned response that promotes fungal survival across diverse host niches [44,45]. These findings underscore the need for deeper molecular studies on the regulation of Sap expression to support the development of more effective, targeted antifungal therapies.

## 5. Conclusions

This study demonstrated that Sap production in species of the *C. haemulonii* clade is strongly influenced by environmental factors, with optimal enzymatic activity occurring at acidic pH values (4.0 and 5.0) and at 37 °C, which are conditions that closely resemble the human physiological environment. These findings suggest that Sap expression is finely tuned to host-related conditions, potentially contributing to fungal adaptation and pathogenicity. Furthermore, the evaluation of various protein substrates revealed that BSA, HSA and globulin were the most effective in inducing Sap activity. Their abundance in human tissues and bloodstream reinforces their physiological relevance and supports their use as model substrates in the investigation of fungal virulence mechanisms. Despite these general patterns, the heterogeneity observed among clinical isolates highlights that Sap regulation likely occurs in a species- and isolate-specific manner, possibly reflecting genetic or epigenetic variation. Altogether, these findings underscore the adaptive capacity of *C. auris* and members of the *C. haemulonii* species complex. They also reinforce the importance of future molecular investigations to unravel the regulatory pathways controlling Sap expression and to clarify its contributions to pathogenicity, immune evasion and resistance to antifungal agents.

## Figures and Tables

**Table 1:** Epidemiological and antifungal susceptibility profiles of clinical isolates of *Candida auris* and the *Candida haemulonii* complex used in this study.

*Candida* Species	Isolate Codes	Origin	Country	Year of Collection	Isolation Site	Antifungal Susceptibility Profile	
						FLC	AMB
*C. auris*	Ca885	CIMCE	Colombia	2016	Blood	R	S
*C. auris*	Ca446	UJ	Colombia	2016	Blood	R	S
*C. auris*	Ca383	CDC	USA	2012	Blood	R	S
*C. auris*	Ca384	CDC	USA	2012	Blood	R	S
*C. haemulonii* var. *vulnera*	Ch11	INCA	Brazil	2013	Blood	R	R
*C. haemulonii*	Ch12	INCA	Brazil	2013	Blood	R	R
*C. duobushaemulonii*	Ch13	DASA	Brazil	2012	Blood	R	R
*C. duobushaemulonii*	Ch23	DASA	Brazil	2011	Blood	R	R

FLC—Fluconazol; AMB—Amphotericin B; R—Resistant; S—Susceptible. CIMCE—Centro de Investigaciones Microbiológicas del Cesar, UJ—Universidad Javeriana, CDC—Centers for Disease Control and Prevention, INCA—Instituto Nacional do Câncer do Rio de Janeiro, DASA—Diagnósticos da América S.A.

## Data Availability

The original contributions presented in this study are included in the article. Further inquiries can be directed to the corresponding author.
